# Serum Oxytocin Levels Decrease 12 Months Following Sleeve Gastrectomy and Are Associated with Decreases in Lean Mass

**DOI:** 10.3390/ijms241210144

**Published:** 2023-06-14

**Authors:** Imen Becetti, Vibha Singhal, Supritha Nimmala, Hang Lee, Elizabeth A. Lawson, Miriam A. Bredella, Madhusmita Misra

**Affiliations:** 1 Division of Pediatric Endocrinology, Mass General for Children and Harvard Medical School, Boston, MA 02114, USA; mmisra@mgh.harvard.edu; 2 Neuroendocrine Unit, Massachusetts General Hospital and Harvard Medical School, Boston, MA 02114, USA; supritha92@gmail.com (S.N.); ealawson@mgh.harvard.edu (E.A.L.); 3 Pediatric Program, MGH Weight Center, Massachusetts General Hospital, Boston, MA 02114, USA; 4 Biostatistics Center, Massachusetts General Hospital, Boston, MA 02114, USA; hlee5@mgh.harvard.edu; 5 Department of Radiology, Musculoskeletal Imaging and Interventions, Massachusetts General Hospital and Harvard Medical School, Boston, MA 02114, USA; mbredella@mgh.harvard.edu

**Keywords:** oxytocin, obesity, sleeve gastrectomy, youth, lean mass

## Abstract

Oxytocin (OXT), an anorexigenic hormone, is also bone anabolic. Further, OXT administration results in increases in lean mass (LM) in adults with sarcopenic obesity. We examine, for the first time, associations of OXT with body composition and bone endpoints in 25 youth 13–25 years old with severe obesity who underwent sleeve gastrectomy (SG) and 27 non-surgical controls (NS). Forty participants were female. Subjects underwent fasting blood tests for serum OXT and DXA for areal bone mineral density (aBMD) and body composition. At baseline, SG vs. NS had higher median body mass index (BMI) but did not differ for age or OXT levels. Over 12 months, SG vs. NS had greater reductions in BMI, LM, and fat mass (FM). OXT decreased in SG vs. NS 12 months post-SG. While baseline OXT predicted a 12-month BMI change in SG, decreases in OXT levels 12 months post-SG were not associated with decreases in weight or BMI. In SG, decreases in OXT were positively associated with decreases in LM but not with decreases in FM or aBMD. Loss of LM, a strong predictor of BMD, after bariatric surgery may reduce functional and muscular capacity. OXT pathways may be targeted to prevent LM loss following SG.

## 1. Introduction

Increasing evidence has supported the role of oxytocin (OXT) in regulating metabolism and body composition. Studies have highlighted the anorexigenic properties of OXT and its role in modulating caloric intake and energy homeostasis [1,2,3,4,5,6,7,8], with recent trials investigating the use of intranasal OXT as a potential therapeutic intervention in the management of obesity [6,7,9,10,11]. Additionally, studies in animal models have demonstrated its necessary role in muscle regeneration [12]. In fact, intranasal OXT administration in older adults with obesity resulted in increases in lean mass, which highlights OXT’s potential use in improving the physical limitations associated with sarcopenic obesity [13]. Furthermore, there has been compelling evidence demonstrating the anabolic effects of peripheral OXT on bone as it stimulates osteoblast differentiation and function [14,15,16,17,18,19,20,21,22,23], thereby positioning it as a potential bone anabolic agent.

Obesity rates in youth have been increasing rapidly. Data from the 2017–2018 National Health and Nutritional Examination Survey estimate that 19.3% of youth have obesity, with 6.1% having severe obesity, defined as a body mass index (BMI) at or above 120% of the 95th percentile for age and sex, and an additional 16.1% being in the overweight category [24]. With increasing rates of severe obesity and the concomitant rise in obesity-related metabolic comorbidities in youth [25], effective weight management interventions, such as metabolic and bariatric surgery (MBS), are increasingly employed to combat these health concerns [26]. MBS, including sleeve gastrectomy (SG) and Roux-en-Y gastric bypass (RYGB), leads to successful weight loss and remission of metabolic comorbidities in youth [27,28,29]. However, the downsides of these interventions include potential weight regain, primarily as fat mass, lean mass reduction, loss of bone mass, and increased long-term skeletal fragility [30,31,32,33,34,35,36,37]. Only a few studies have investigated changes in OXT levels following MBS and have demonstrated conflicting results, with one study showing a significant decrease in OXT levels in adults undergoing gastric banding [38], while another small study found that RYGB had no impact on OXT levels in adults [39].

Given OXT’s anorexigenic effects and bone anabolic properties, we sought to understand the effects of SG (the most commonly used MBS to induce weight loss in adolescents with moderate to severe obesity) on OXT levels in youth. We measured OXT levels in youth with severe obesity undergoing SG or standard therapy and examined associations with weight, body composition, and bone parameters. We hypothesized that OXT levels would decrease following SG, given the decrease in energy stores, and that decreases in OXT would be associated positively with changes in weight, lean mass, and bone endpoints.

## 2. Results

### 2.1. Baseline Characteristics

At baseline, the SG group had higher weight, BMI, and total fat mass compared to the non-surgical control (NS) group; however, the groups did not differ for age, sex, race, BMI z-score, percent fat mass, or total and percent lean mass (Table 1). The use of oral contraceptive pills (OCPs) and serum OXT levels did not differ between groups (Table 1). The groups also did not differ at baseline for areal bone mineral density (aBMD) Z-scores (Table 1).

### 2.2. Changes over 12 Months in Serum OXT Levels

**Figure 1:**
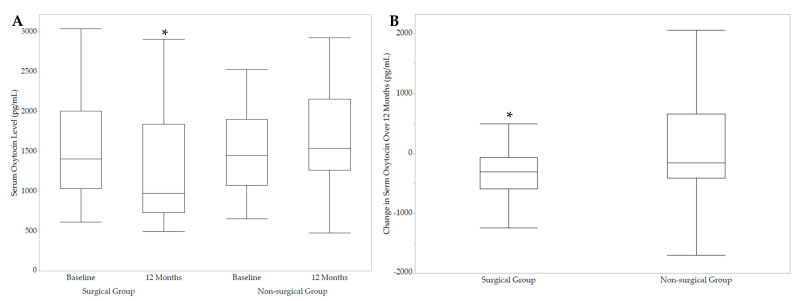
Serum oxytocin levels at baseline and 12 months in the surgical and non-surgical groups (**A**), and changes in serum oxytocin levels over 12 months in the surgical and non-surgical groups (**B**). * *p* < 0.05.

Serum OXT levels significantly decreased in the SG group over 12 months compared to the NS group (*p* = 0.04), with a significant within-group decrease following SG (Table 1, Figure 1). This remained significant after excluding participants taking OCPs at baseline and/or at 12 months. However, after controlling for change in BMI, the change in OXT levels was no longer significantly different between groups.

### 2.3. Changes over 12 Months in Weight and Body Composition

Over 12 months, the SG group had greater reductions in weight, BMI, BMI z-score, and total and percent lean and fat mass (*p* < 0.0001), as shown in Table 1, with significant within-group reductions in these parameters following SG.

### 2.4. Changes over 12 Months in Bone Parameters

As we have previously reported in a slightly different cohort [31], lumbar spine, femoral neck, and total hip BMD Z-scores decreased in the SG vs. NS groups (*p* = 0.04, 0.002, and <0.0001, respectively) with significant within-group reductions. Changes in total body BMD Z-score were not significant within or between groups (Table 1).

### 2.5. Associations between Baseline Serum OXT Levels and Changes in Weight and BMI

In the SG group, baseline serum OXT levels were positively associated with changes in weight (ρ = 0.52, *p* = 0.008) and BMI (ρ = 0.53, *p* = 0.006) over 12 months. However, after excluding participants taking OCPs at baseline, these associations were no longer significant.

### 2.6. Associations between Changes in Serum OXT Levels and Changes in Weight, Body Composition, and Bone Parameters

The reductions in serum OXT experienced in the SG group were not associated with 12-month changes in weight or BMI (Table 2). Decreases in serum OXT levels in the SG group were positively associated with decreases in lean mass; there were no significant correlations between OXT changes and changes in fat mass (Table 2). Serum OXT changes over 12 months were negatively associated with changes in lumbar BMD Z-score (Table 2). After controlling for changes in BMI and lean mass, the association of change in OXT with change in lumbar BMD Z-score was no longer significant.

## 3. Discussion

Our data demonstrate that adolescents and young adults with severe obesity had a significant reduction in serum OXT levels 12 months following SG compared to those receiving standard therapy, primarily mediated by reductions in BMI. The SG group demonstrated expected reductions in weight, BMI, lean mass, fat mass, and body composition, as well as changes in dual-energy x-ray absorptiometry (DXA) endpoints that have been previously reported [31]. Baseline serum OXT levels were positively associated with changes in weight and BMI over 12 months; however, this association was no longer significant after excluding participants on OCPs. Additionally, changes in serum OXT levels were positively associated with changes in lean mass. Associations with changes in lumbar BMD Z-score were not significant after controlling for changes in BMI and lean mass. To our knowledge, this is the first report of changes in serum OXT levels following SG in youth and associations with body composition and bone endpoints over 12 months.

Data from our cohort demonstrate reductions in serum OXT levels following SG in youth with severe obesity. This remained significant after excluding participants taking OCPs at baseline and/or at 12 months. However, this reduction was no longer significant after controlling for changes in BMI, which supports our hypothesis that serum OXT levels reflect energy availability, with higher levels seen in individuals with higher BMI than in normal-weight individuals (41). The reduction in serum OXT levels is also consistent with a prior study showing similar findings in adults undergoing gastric banding [38], although another small study reported no changes in OXT levels in adults undergoing RYGB [39].

While baseline serum OXT levels were not associated with baseline weight or BMI in our cohort (nor the 12-month OXT levels associated with 12-month weight or BMI), we observed a positive association between baseline serum OXT levels and changes over 12 months in weight and BMI following SG. However, this association was no longer significant after excluding participants using OCPs at baseline. Prior studies in humans have demonstrated increases in OXT levels in response to oral estrogen administration (such as with the use of OCPs) [40,41]. Therefore, these associations between baseline OXT levels and weight or BMI changes over 12 months appear to have been driven by higher OXT levels in participants using OCPs and were no longer present after excluding these participants.

In this study, we observed a positive association between changes in serum OXT levels and lean mass over 12 months. Lean mass loss has been linked to reduced muscular and functional capacity [36,42], which carries significant implications for the long-term quality of life of individuals undergoing SG. Additionally, lean mass is a strong predictor of BMD, as higher lean mass has been associated with higher BMD in youth [43,44,45]. Therefore, lean mass loss may be implicated in the loss of BMD previously demonstrated in our cohort [31,32]. The observed positive association between serum OXT levels and lean mass is also consistent with data indicating that administration of intranasal OXT results in increases in lean mass in older adults with sarcopenic obesity [13]. Our findings suggest that OXT administration may prevent the expected lean mass loss after SG with beneficial effects on long-term bone health and quality of life. Future studies investigating such interventions are necessary to evaluate these effects, as well as energy expenditure outcomes in relation to oxytocin levels and lean mass changes.

Lastly, changes in serum OXT levels were negatively associated with changes in lumbar BMD Z-scores over 12 months in our cohort. This association is not consistent with prior studies demonstrating OXT’s anabolic properties on bone and its positive relationship with BMD [14,15,16,18,19,20,21,22,23]. This association may have arisen by chance. Additionally, after controlling for changes in BMI and lean mass, changes in serum OXT levels were no longer associated with changes in lumbar BMD Z-scores, suggesting that the observed association was driven by changes in BMI and lean mass rather than reductions in serum OXT levels.

Our study has limitations due to the relatively small number of participants undergoing SG as well as the short duration of follow-up. Future studies with a larger cohort of participants and a longer follow-up duration are necessary to further characterize changes in serum OXT levels after SG. Additionally, the demonstrated associations do not prove causation, given the observational nature of our study. Further, we did not control for multiple comparisons as this is an exploratory study. Our study also has many strengths, such as the inclusion of a comparison group of non-surgical controls not available in other studies. Our study sheds light on the effects of SG on serum OXT levels in adolescents and young adults with obesity, a sparse area of research.

In conclusion, our study demonstrates decreases in OXT levels following SG in youth. Reductions in OXT levels are positively associated with reductions in lean mass following SG. Future studies are necessary to understand how OXT can be manipulated to prevent this loss in lean mass, such as administering intranasal oxytocin post-operatively and examining whether it can maintain lean muscle mass.

## 4. Materials and Methods

### 4.1. Participant Selection

Fifty-two adolescents and young adults aged 13–25 years old with moderate to severe obesity were enrolled in this study. Twenty-five participants (19 female and 6 male) underwent SG, while 27 (21 female and 6 male) were non-surgical controls who received standard medical therapy. Inclusion criteria included (i) a BMI of ≥35 kg/m^2^ with obesity-related complications or a BMI ≥ 40 kg/m^2^ to meet requirements for MBS, and (ii) for the surgical group, a plan to undergo sleeve gastrectomy (a decision made by the treating physician). Exclusion criteria included (i) females currently pregnant or breastfeeding, (ii) use of medications that affect bone, such as oral glucocorticoids, phenytoin, or phenobarbitone within 8 weeks of the baseline visit (participants on calcium or vitamin D supplements, OCPs, or progesterone were not excluded because of the frequency of their use and to ensure that we had a representative sample), (iii) use of antipsychotic medications associated with weight gain if treatment duration was less than 6 months or if the dose was adjusted within 2 months prior to the baseline visit, (iv) uncontrolled thyroid disease or adjustment of replacement levothyroxine within 3 months prior to the baseline visit, (v) history of substance abuse per DSM-5 criteria or smoking (>10 cigarettes/day, and (vi) history of radiation exposure greater than 10 millisieverts (mSv) in the 12 months prior to enrollment.

Participants were recruited from several tertiary care obesity treatment centers focused on lifestyle and surgical interventions for weight management. The study was approved by the Partners Institutional Review Board and was compliant with the Health Insurance Portability and Accountability Act. Participants ≥ 18 years and parents of participants < 18 years provided written informed consent. Participants < 18 years provided informed assent.

### 4.2. Study Visits

A screening visit was first performed to confirm eligibility for the study. Study visits were performed at baseline (within a month prior to sleeve gastrectomy) and 12 months after sleeve gastrectomy. Non-surgical control subjects were also examined at baseline and then after 12 months. A medical history, physical examination, and anthropometric measurements were obtained from all participants. Weight was measured to the nearest 0.1 kg using an electronic scale, and height was measured using a wall-mounted stadiometer as the mean of three measurements. BMI was calculated as weight in kg/(height in meters)^2^. Each subject underwent fasting blood tests for serum OXT and DXA for aBMD and body composition at baseline and 12 months. Non-surgical controls received standard diet and exercise counseling throughout the study from their primary care provider, specialized programs they were enrolled in, or dieticians from our Translational and Clinical Research Center. Surgical and non-surgical participants received vitamin D and calcium supplementation as previously described [31]. All participants were offered a minimum of 800 international units (IUs) of vitamin D and 1200 mg of elemental calcium daily to enhance calcium intake and absorption. Additional supplementation was advised based on 25(OH) vitamin D (25OHD) levels and recommendations typically provided for those undergoing surgery [46,47]: for 25OHD levels between 20 and 30 ng/mL: 4000 IUs of vitamin D supplementation daily; for 25OHD levels between 12 and 20 ng/mL: 50,000 IUs of vitamin D per week for two months followed by 2000 IUs daily; for 25OHD levels <12 ng/mL: 50,000 IUs of vitamin D per week for three months followed by 2000 IUs daily.

### 4.3. Biochemical Analysis

Serum OXT levels were measured in unextracted serum by Enzyme-Linked Immunoassay (ELISA) (Enzo Life Sciences, Farmingdale, NY, USA), with intra-assay and inter-assay coefficient of variation (CV) of 10.2–13.3% and 11.8–20.9%, respectively. The detection limit was 15 pg/mL. Extracted and unextracted serum OXT levels were previously demonstrated to have a strong correlation [14].

### 4.4. Dual Energy X-ray Absorptiometry (DXA)

DXA (Hologic 4500 A, Waltham, MA, USA) was used to assess aBMD of the lumbar spine, total hip, femoral neck, and whole body, as previously described [31]. DXA was also used to assess total body lean and fat mass.

### 4.5. Statistical Analysis

JMP Statistical Discovery Software (Version 16, SAS Institute, Carey, NC, USA) was used to perform all statistical analyses. Baseline characteristics and 12-month changes between SG and NS groups were compared by Student’s *t*-test or the Wilcoxon Rank Sum test, depending on data distribution. Similarly, within-group comparisons were assessed using paired *t*-tests or the Wilcoxon sign rank test depending on data distribution. General linear model (GLM) was used to determine differences between groups after controlling for possible covariates such as baseline or 12-month change in BMI. To determine associations between OXT changes and BMI, body composition, and aBMD, linear regression analyses were performed. Spearman’s correlation coefficients are reported. *p* ≤ 0.05 was used to denote significance. Data are presented as mean ± standard error of the mean (SEM) or median (interquartile range) unless otherwise indicated.

## Figures and Tables

**Table 1:** Baseline characteristics and changes over 12 months in clinical characteristics, OXT, body composition, and bone parameters for sleeve gastrectomy (surgical) and non-surgical groups.

	Baseline Measure		Change over 12 Months		*p*-Value Comparing Changes over 12 Months across Groups
	Surgical (n = 25)	Non-Surgical (n = 27)	Surgical (n = 25)	Non-Surgical (n = 27)	
**Clinical Characteristics**					
Age (years)	18.15 ± 0.42	18.22 ± 0.56	-	-	-
Sex (Female/Male)	19/6	21/6	-	-	-
Race (Black/Other/White)	6/4/15	5/8/14	-	-	-
Weight (kg)	132.00 ± 5.20	117.70 ± 4.00 *	−32.54 ± 2.25 *	0.95 ± 1.72	**<0.0001**
BMI (kg/m^2^)	44.96 (42.23, 50.41)	41.60 (37.86, 46.41) *	−11.47 (−14.48, −8.57) *	0.92 (−1.31, 1.77)	**<0.0001**
BMI z-score	2.50 (2.34, 2.78)	2.38 (2.19, 2.66)	−0.62 (−0.96, 0.41) *	−0.03 (−0.10, −0.00)	**<0.0001**
**Serum OXT**	1407.00 (1034.50, 2010.50)	1449.00 (1075.00, 1903.00)	−311.00 (−596.00,−69.50) *	−160.00 (−411.00, 651.00)	**0.04**
**Body Composition**					
Total lean mass (kg)	63.55 ± 2.09	60.54 ± 2.08	−9.37 ± 0.81 *	1.91 ± 0.59	**<0.0001**
% Lean mass	49.07 ± 0.87	50.94 ± 0.83	7.19 ± 1.06 *	1.27 ± 0.57	**<0.0001**
Total fat mass (kg)	63.73 ± 2.44	56.13 ± 2.28 *	−23.25 ± 2.07 *	−0.91 ± 1.33	**<0.0001**
% Fat mass	49.02 ± 0.88	47.11 ± 0.85	−7.83 ± 1.11 *	−1.23 ± 0.57	**<0.0001**
**Areal BMD Z-score**					
Lumbar BMD	1.10 (0.50, 1.50)	0.90 (0.50, 1.60)	−0.40 (−0.60, 0.00) *	0.00 (−0.30, 0.23)	**0.04**
Femoral neck BMD	1.45 (0.95, 1.90)	1.60 (0.80, 2.30)	−0.80 (−1.25, −0.30) *	−0.05 (−0.40, 0.25)	**0.0002**
Total Hip BMD	1.50 (0.68, 2.00)	1.70 (0.80, 2.20)	−0.85 (−1.30, −0.40) *	−0.10 (−0.23, 0.10)	**<0.0001**
Total Body BMD	−0.20 (−0.90, 0.40)	−0.20 (−0.80, 0.80)	0.00 (−0.30, 0.50)	0.00 (−0.45, 0.20)	0.47

* *p* < 0.05 for within-group comparisons. Significant *p*-values (<0.05) are bolded for between-group significance. Abbreviations: BMD—Bone mineral density; BMI—body mass index; OXT—oxytocin. Means ± SEM or Median (interquartile range) are reported. Significant changes from baseline and significant *p* values are bolded. Baseline measures and differences between groups were compared using the Student *t*-test for parametric data and the 2-sample Wilcoxon rank sum test for non-parametric data; within-group changes over 12 months were assessed using the paired test-test for parametric data, and the Wilcoxon sign rank test for non-parametric data.

**Table 2:** Associations of changes in serum OXT with changes in weight, body composition, and bone parameters over 12 months in the sleeve gastrectomy group.

Variable	12-Month Change in Serum OXT		
12-Month Change	ρ	*p*-Value	*p*-Value after Controlling for Change in BMI and Lean Mass
Weight	0.08	0.71	-
BMI	0.09	0.66	-
Lean Mass	0.50	**0.01**	-
Fat Mass	0.003	0.99	0.70
Lumbar BMD Z-score	−0.49	**0.02**	0.63
Femoral neck BMD Z-score	0.15	0.50	0.23
Total Hip BMD Z-score	0.13	0.56	0.38
Total Body BMD Z-score	−0.15	0.48	0.69

Spearman correlations (ρ) and the corresponding *p*-values are reported. Significant *p*-values are bolded. Abbreviations: BMD—Bone mineral density; BMI—body mass index; OXT—oxytocin.

## Data Availability

The data are not publicly available as the study is still ongoing.
